# Using pension payments abroad to examine post-retirement migration and health among Finnish migrants in Sweden

**DOI:** 10.1016/j.jmh.2026.100411

**Published:** 2026-03-31

**Authors:** Agneta Cederström, Kaarina Korhonen, Pekka Martikainen, Olof M Östergren

**Affiliations:** a Stockholm University, Department of Public Health Sciences, SE - 106 91 Stockholm, Sweden; b Stockholm University/Karolinska Institutet, Centre for Health Equity Studies (CHESS), SE-106 91 Stockholm, Sweden; c University of Helsinki, Helsinki Institute for Demography and Population Health, P.O. Box 3 Unioninkatu 33, 00014 Helsinki, Finland; d University of Helsinki, Max Planck–University of Helsinki Center for Social Inequalities in Population Health, P.O.Box 59, FI-00014 Helsinki, Finland

**Keywords:** Post-retirement migration, Return migration, Migrant health, Health selection, Salmon bias, Pension payments

## Abstract

This prospective cohort study used linked Swedish administrative registers and pension payment data to examine post-retirement migration patterns and health selection among Finnish migrants compared to native-born Swedes. The cohort included individuals born between 1938 and 1955 who were resident in Sweden at age 64, with follow-up from age 65 until death or the end of 2021 (*n* = 1794,196; 80,313 Finnish-born). Health status was assessed using the Charlson Comorbidity Index (CCI), while pension payments were used to identify country of residence and mortality occurring after emigration. Post-retirement emigration was substantially more common among Finnish migrants (3.0 %) than native-born Swedes (0.3 %), primarily due to return migration to Finland. Contrary to the “salmon bias” hypothesis, poorer health reduced rather than increased the likelihood of emigration for both groups. Among Finnish migrants, lower CCI scores, male sex, being unmarried, and lower income predicted a higher likelihood of post-retirement migration. Sequence analyses revealed three distinct Finnish migration trajectories: early return, late return, and return with onward migration. Mortality did not differ significantly between Finnish migrants who remained in Sweden and those who resided in Finland or other countries; adjusted models even indicated slightly lower mortality among return migrants Extending follow-up abroad with pension data did not change mortality estimates, indicating unrecorded deaths abroad likely did not bias results. The findings indicate positive health selection into return migration and no evidence of a salmon bias effect. Post-retirement migration among Finnish migrants in Sweden is rare and has little impact on migrant–native mortality differences.

## Highlights

• Finnish migrants in Sweden were more likely than natives to emigrate after retirement, mostly returning to Finland.
• Poor health reduced post-retirement emigration in both migrants and natives supporting the healthy migrant hypothesis.
• Among Finnish migrants, better health, male sex, being unmarried, and lower income predicted post-retirement migration.
• Mortality was similar for return migrants compared to those staying in Sweden, but slightly lower after adjusting for SES.
• Pension data offer a novel way to track migration and mortality after emigration, reducing bias from deaths abroad..

## 1 Introduction

Migration in later life is a complex phenomenon influenced by health, socioeconomic status, and personal circumstances. While migration is often associated with early adulthood (Bentham, 1988), post-retirement migration has received increasing attention, particularly in relation to global trends in population aging and transnational mobility (Maleku et al., 2022; Savaş et al., 2023). In this study, we investigate the relationship between health, sociodemographic conditions and post-retirement migration patterns among Finnish-born and Swedish-born individuals residing in Sweden at retirement age.

Selection is an inherent issue when trying to understand populations that move across borders, and therefore in and out of national administrative data. The "salmon bias" hypothesis suggests that migrants in poor health are more likely to return to their country of origin before death, potentially leading to an underestimation of migrant mortality in host countries (Di Napoli et al., 2021; Abraído-Lanza et al., 1999; Turra and Elo, 2008). Conversely, the "healthy migrant effect" posits that migrants tend to be positively selected for health at the time of migration, which helps explain their observed mortality advantage compared to the host population (Aldridge et al., 2018; Shor and Roelfs, 2021). This advantage is often most pronounced among migrants from the Global South to the Global North, those who have migrated over long distances, or individuals belonging to visible minority groups, whereas the migrant mortality advantage tends to be smaller—or even reversed—for those arriving from neighboring countries (Roelfs and Shor, 2022). These mortality advantages persist despite migrants lower socioeconomic status in the host country; a phenomenon often referred to as the migrant mortality paradox (Markides and Rote, 2015). Nevertheless, empirical evidence on these patterns remains mixed, particularly for older migrants in high-income economies.

There have been two general approaches to empirically evaluate health selection in relation to return migration. Some studies estimate the risk of emigration from the country of destination by indicators of comorbidity and healthcare utilization**,** whilst others use administrative data to track mortality after emigration with results varying across contexts. Negative health selection in return migration is found in Mexican migrants in the United States, both in longitudinal survey data (Arenas et al., 2015), and in analyses of Social Security data (Turra and Elo, 2008)^.^ In addition, French pension data indicate that return migrants have higher mortality, especially at younger ages (Guillot et al., 2023). In contrast, evidence from Germany suggests that return migration among Turkish migrants is more likely among men in better health with no discernible effect among women, but also that factors such as family and economic considerations are likely more important in return decisions (Razum et al., 2005; Sander, 2007). Similarly, register-based studies in Nordic countries find that healthier migrants are more likely to remigrate (Dunlavy et al., 2022; Handlos et al., 2018; Norredam et al., 2014). Internal migration studies also demonstrate heterogeneity: studies on migrants between England and Scotland and internal migrants in the Netherlands found that migrants were generally healthier than non-migrants (Puschmann et al., 2017; Wallace and Kulu, 2014), with no evidence that poor health predicted return, while research on rural–urban migration in China reports both healthy-migrant selection and some return of less healthy individuals to origin communities (Lu and Qin, 2014). Overall, these findings suggest that negative health selection in return migration is highly context-dependent, varying by migrant type, age, and access to health care. However, in settings where negative health selection in return migration was observed, the mortality differences and rate of remigration were not large enough to explain the migrant mortality advantage.

In the present study, we combine both approaches to examine mortality patterns among Finnish migrants in Sweden. Finnish migrants to Sweden mostly arrived as labor migrants in the late 1960s and early 1970s and unlike other more recently arrived migrant groups, exhibit a mortality disadvantage relative to the native-born population, primarily due to higher alcohol- and cardiovascular disease (CVD)-related mortality (Östergren et al., 2021). We use hospitalization records to estimate health status as a predictor of emigration, and pension payment records to track mortality risks after emigration. In addition, using pension payment records, we can identify post-retirement migration trajectories, their socioeconomic predictors, and distinguishing between return migration to Finland and onward migration to other countries. This latter distinction is important as the salmon bias hypothesis specifically implies return migration rather than onward migration.

This study contributes to the literature on later-life migration and migrant health by providing novel insights into whether return and onward migration are health-selective processes. Additionally, we assess whether mortality differentials exist between those of Finnish origin who remain in Sweden and those who migrate after retirement. We also leverage the detailed nature of pension payments to uncover typical post-retirement migration trajectories and their sociodemographic predictors in both the Finnish migrant and native-born populations. Our findings have implications for understanding how aging and migration intersect with health in an increasingly globalized world marked with rising rates of migration.

## 2 Materials and methods

### 2.1 Data

We use several linked administrative registers covering the total population of Sweden. The study population comprises all persons born in Finland and Sweden between 1938 and 1955, alive in 2003, and living in Sweden at the age of 64, the legal age of retirement. The population is followed for residential status and mortality until 2021.

We use the national inpatient register to calculate the Charlson Comorbidity Index (CCI) (Charlson et al., 1987) which was constructed as a predictor for long-term mortality. The CCI is assessed each year an individual is resident in Sweden and used as a time-varying measure of health status prior to emigration. In analyses where the exposure is time-varying country of residence the CCI is included at the age of 65 since it cannot be measured after emigration.

We measure mortality using the cause of death register for individuals residing in Sweden and data on pension payments abroad to track mortality after emigration. All individuals have the right to receive a pension from labour market activity, including widows’ pension, even if the individual emigrates from Sweden. In order to keep receiving the payments, the individual submits a certificate of life to verify that they are still alive. We obtain data from the Swedish Pension Agency on all monthly pension payments from Sweden to abroad during 2003–2021 and use the last payment to proxy the month of death. Some countries, including Finland, have an agreement with Sweden where the authorities will inform the Swedish pension agency if a recipient has died. For these deaths, we used the exact date of death.

We also obtain information on the country of residence, proxied by the country to which the pension payment was sent for each monthly payment. For integrity reasons, we received this data in truncated format and classified it into two categories, Finland (return migration for Finnish migrants) and Other (onwards migration). In some cases, there were pension payments sent to both Sweden and a second country in the same month. In these cases, the person was assumed to live in Sweden if the person was officially registered at an address in Sweden, or else they were classified as living in the other country where the payment was made.

**Fig. 1:**
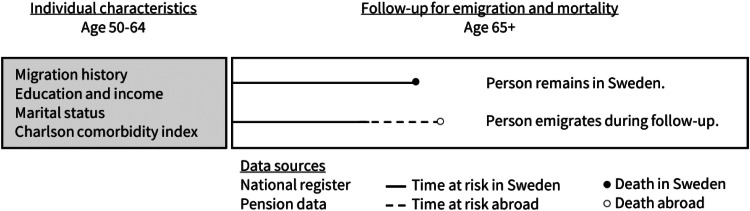
Data structure showing how follow-up is extended post-emigration with the use of pension payments.

We link data from other administrative registers containing demographic characteristics and socioeconomic position. From Statistics Sweden, we obtain annual information on income, education, and marital status. Income is defined as the mean income between 50–60 years of age adjusted for inflation and is divided into quintiles. We categorize education into compulsory, intermediate or tertiary and marital status into married/not married. Education and marital status are assessed at age 64. We use the multi-generational register to assess the number of children in Sweden (0, 1, 2 or more). Fig. 1 outlines the data structure.

### 2.2 Statistical analysis

To empirically assess the salmon bias hypothesis, we fit a Poisson model to estimate incidence rate ratios (IRR) for the occurrence of events over person-time during follow-up with 95 % confidence intervals (CI) where emigration from Sweden is the event and the Charlson Comorbidity Index (CCI) serves as the predictor. Given that our outcome variable is binary, we estimated the Poisson regression model with robust standard errors to account for potential overdispersion and ensure consistent parameter estimates. Demographic and socioeconomic factors influencing post-retirement emigration are also included. Follow-up begins at retirement and ends upon emigration, death in Sweden, or right-censoring at the study's conclusion. The models are stratified by country of birth. Model 1 (M1) adjust for age, sex, marital status, number of children, education, and income. Model 2 (M2) additionally adjusts for duration of residence for the Finnish migrants. Additionally, we leverage pension record data and use Poisson models with robust standard errors to estimate mortality rate ratios (MRR) where death is the event. The salmon bias hypothesis is tested by comparing models right-censoring at emigration and models which extend follow-up abroad using pension data. In order to specifically examine return migration, we then run models which treat country of residence (Sweden, Finland, or Other) as a time-varying exposure, and interact this with country of birth. This approach enables us to assess whether mortality risk varies systematically among those who remained in Sweden, migrated back to Finland, or moved to a third country after retirement. We also include an interaction between country of birth and country of residence to determine whether mortality risk differs between Finnish migrants and native-born individuals across residence locations. Model 1 is adjusted for age, sex, and calendar year, and an interaction between country of birth and country of residence. Model 2 is additionally adjusted for CCI at 64, marital status, education, occupational status, and number of children. Age and calendar year are included as separate covariates to distinguish the effects of chronological aging from secular trends, as individuals enter the study at different ages and calendar years.

In order to further refine the analyses and use all the information contained in the pension data, we use sequence analyses to analyze post-retirement migration patterns, stratified by country of birth (Finland or Sweden). Sequences begin at the age of 65 and stop at death or end of follow up and uses age as the time scale. The states were categorized by country of residence and included Sweden (S), Finland (F), and Other (O) country which allows us to distinguish between return and onward migration for Finnish migrants residing in Sweden at retirement age. Residents that remained in Sweden during the whole follow-up were identified as “Stay in Sweden” and were not included in the clustering analysis. The remaining country of residence sequences were clustered using the Optimal Matching algorithm using estimated INDELSLOG for the substitution matrix. The optimal number of clusters were investigated with several measures, including the average silhouette width. Each individual was then assigned membership to a cluster. Cluster membership was considered as an outcome in a multinomial regression with CCI assessed at age 64 as exposure to analyze health selection into post-retirement migration patterns.

## 3 Results

The study population included 1794,196 individuals of which 80,313 (4.5 %) were born in Finland. Of the total study population, 7418 (0.4 %) emigrate post-retirement, out of which 2270 (30.6 %) migrated to Finland, the majority of which are Finnish-born migrants returning to Finland (91.7 %). Table 1 shows the total number of individuals, the number of individuals remaining in Sweden or emigrating to other countries, and the number of deaths for the Finnish and Swedish-born populations. Deaths are included to illustrate that pension data allow tracking of deaths both in Sweden and abroad.

**Table 1:** Migration patterns and deaths among Swedish natives and Finnish migrants.

	Finnish migrants	Native-born
**Individuals**	**N (%)**	**N (%)**
Total	80,313	1713,833
Emigrated (total)	2388 (3.0)	5030 (0.3)
Emigrated (Finland)	2087 (2.6)	188 (0.01)
Emigrated (Other)	307 (0.4)	4844 (0.3)
**Deaths**	**N (%)**	**N (%)**
Total	15,553	261,095
Finland	410 (2.6)	23 (0.01)
Other	51 (0.4)	511 (0.2)

Note: Some individuals may appear in both Finland and Other countries if they emigrated to both during follow-up.

Although Finnish-born individuals are more likely to emigrate after retirement than Swedish-born individuals (3.0 % vs. 0.3 %), the vast majority of Finnish migrants remain in Sweden (97 %). The higher emigration rate among the Finnish-born is primarily driven by return migration to Finland (2.6 %), whereas relocation to other countries occurs at similar levels among Finnish migrants (0.4 %) and Swedish-born individuals (0.3 %).

### 3.1 Health selection into emigration

Table 2 shows the estimated IRR and 95 % CI obtained from Poisson regressions with emigration as outcome and underlying health (CCI) as exposure. Age, sex, country of birth, income, education, marital status, and number of children are included in the Model 1 (M1) while duration of residence was additionally included in Model 2 (M2) for Finnish migrants.

**Table 2:** Demographic, socioeconomic and health-related predictors of post retirement migration among Finnish migrants and the native-born. Incidence rate ratios (IRR) are estimated using stratified Poisson regressions with post-retirement emigration from Sweden as the event with a 95 % confidence interval (CI). Model 1 (M1) adjust for age, sex, marital status, number of children, education, income, and Charlson comorbidity index. Model 2 (M2) additionally adjusts for duration of residence for the Finnish migrants.

	**Finnish migrants**	**Native-born**	**Finnish migrants**
	M1: IRR (95 % CI)	M1: IRR (95 % CI)	M2: IRR (95 % CI)
**Charlson comorbidity index**			
None	1(ref)	1(ref)	1(ref)
Low	0.45 (0.36–0.57)*	0.31 (0.25–0.39)*	0.44 (0.35–0.57)*
High	0.37 (0.22–0.61)*	0.26 (0.16–0.43)*	0.37 (0.22–0.62)*
**Age**			
Linear	0.92 (0.91–0.93)*	0.87 (0.86–0.88)*	0.93 (0.92–0.94)*
**Sex**			
Male (ref)	1(ref)	1(ref)	1(ref)
Female	0.66 (0.60–0.72)*	0.45 (0.43–0.48)*	0.66 (0.60–0.72)*
**Marital status**			
Not married	1(ref)	1(ref)	1(ref)
Married	0.85 (0.77–0.93)*	0.75 (0.70–0.80)*	0.81 (0.74–0.89)*
**Number of children**			
0	1(ref)	1(ref)	1(ref)
1	0.43 (0.38–0.48)*	1.18 (1.06–1.31)*	0.52 (0.46–0.59)*
2+	0.30 (0.27–0.33)*	0.97 (0.88–1.07)	0.38 (0.34–0.42)*
**Education**			
Compulsory	1(ref)	1(ref)	1(ref)
Intermediate	0.82 (0.75–0.90)*	1.59 (1.47–1.73)*	0.85 (0.77–0.93)*
Tertiary	1.14 (1.00–1.29)*	2.47 (2.24–2.72)*	1.02 (0.90–1.16)
**Disposable income**			
Q1(lowest)	1(ref)	1(ref)	1(ref)
Q2	0.74 (0.66–0.83)*	0.53 (0.48–0.59)*	0.83 (0.74–0.93)*
Q3	0.70 (0.62–0.79)*	0.44 (0.39–0.49)*	0.81 (0.72–0.91)*
Q4	0.65 (0.57–0.74)*	0.51 (0.46–0.57)*	0.76 (0.66–0.87)*
Q5 (highest)	0.72 (0.61–0.84)*	0.96 (0.87–1.06)	0.83 (0.70–0.99)*
**Duration of residence**			
0–15			1(ref)
16–30			0.56 (0.42–0.74)*
30+			0.13 (0.10–0.17)*

⁎ represents statistical significance within *p* < 0.05.

Emigration was predicted by underlying health status, but contrary to the salmon bias effect, those in poor physical health were less likely to emigrate (IRR[95 %CI] Finnish migrants M1 CCI Low: 0.45 [0.36–0.57], CCI High: 0.37 [0.22–0.61] relative to Finnish migrant at zero CCI score; Native-born M1 CCI Low: 0.31 [0.25–0.38], CCI High: 0.26 [0.16–0.43] relative to native born with a zero CCI score). The risk of emigration was lower for women compared to men in both the Finnish migrant (IRR[95 %CI] M1: 0.66 [0.60–0.72])and native-born population men (IRR[95 %CI] M1: 0.45 [0.43–0.48]), The likelihood of post-retirement migration varied independently by several additional demographic and socioeconomic factors. Migration was more likely at younger ages, among men, and for the unmarried; effects which were more pronounced for the native-born. Considering children, education, and income we see that emigration patterns varied by country of birth. Having children in Sweden reduced the propensity for post-retirement emigration for Finnish migrants but not for the native-born. Education effects differed: Finnish migrants with compulsory or tertiary education had higher emigration risk than those with intermediate education, while the native-born showed a linear trend—higher education is associated with higher emigration risks. Low-income residents were most likely to emigrate in both groups, but high-income native-born also had a high propensity to leave. Thus, lower socioeconomic status was less of a barrier for emigration for Finnish migrants compared to the native-born, while having familial ties was more of a barrier for the Finnish migrants. Additionally for the Finnish migrants, longer duration of residence, or equivalently, arrival to Sweden at younger ages, lowered the risk of post-retirement emigration.

### 3.2 Salmon bias

Table S1 shows comparisons between mortality rate ratios when post-retirement follow-up is right censored at emigration and when a follow-up until death is included using pension payment data. The estimates are identical up to the first two decimals showing that unrecorded deaths occurring post-migration do not contribute to mortality differentials between Finnish migrants and native Swedes. In Table 3, we present the estimated mortality rate ratios using country of residence as a time-varying covariate.

**Table 3:** Mortality rate ratio (MRR) with a 95 % CI by post-retirement country of residence among Finnish migrants and the native-born. Model 1 is adjusted for age, sex, and calendar year, and an interaction between country of birth and country of residence. Model 2 is additionally adjusted for CCI, marital status, education, occupational status, and number of children. Estimates for other variables not shown in table.

**Model 1**	MRR (95 % CI)	MRR (95 % CI)
*Interaction with country of birth*		
Country of residence	Finnish migrants	Native-born
Sweden	1 (ref)	1 (ref)
Finland	1.00 (0.91–1.11)	1.03 (0.69–1.53)
Other	1.15 (0.88–1.50)	0.97 (0.89–1.06)

**Model 2**	MRR (95 % CI)	MRR (95 % CI)

*Interaction with country of birth*		
Country of residence	Finnish migrants	Native-born
Sweden	1 (ref)	1 (ref)
Finland	0.91 (0.82–1.00)*	1.02 (0.68–1.53)
Other	1.26(0.96–1.65)	1.03 (0.94–1.26)

⁎ represents statistical significance within *p* < 0.05.

Despite the finding that ill-health decreased the propensity for Finnish migrants to return migrate, the Finnish migrants who resided in Finland post-retirement had similar sex and age-adjusted mortality risks (M1 MRR: 1.00 CI: 0.91–1.11) compared to Finnish migrants that remained resident in Sweden. When further adjusting for sociodemographic conditions the mortality risks are lower in migrants who returned to Finland (M2 MRR: 0.91, CI: 0.82–1.00). In all other cases there was no discernible mortality risk differential between those remaining in Sweden and those residing abroad.

### 3.3 Post-retirement migration patterns

**Fig. 2:**
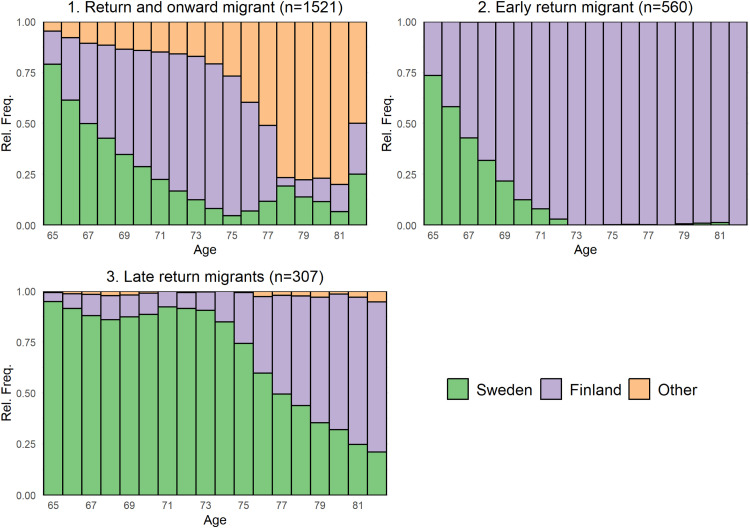
Density plots of the three migration trajectory clusters of country of residence for the Finnish born migrants that emigrated post-retirement. Note: Migrants that remained in Sweden until they died or the end of follow-up (*n* = 77,925) are not represented.

The analyses of post-retirement migration patterns using sequence analyses among the Finnish migrants that emigrated revealed three clusters pictured in Fig. 2. The 77,925 individuals (97.0 %) that remained in Sweden throughout the observation period are not represented in the graph.

The three clusters represent those that 1: return migrated to Finland and onwards to other countries post-retirement (*N* = 1521, 63.7 %), 2: return migrated to Finland within six years of retirement (*N* = 560, 23.5 %), and 3: those that return migrated to Finland after about ten years post-retirement but with few migrations to other countries and some re-return migration to Sweden (*N* = 307, 12.9 %). In Table S2 we present results from a multinomial regression model where cluster membership is the dependent variable and the independent variables are the same socioeconomic, demographic and health-related factors presented in Table 2 (CCI and duration of residence is now assessed at 65 years of age). The results confirm a positive health selection, with ill-health decreasing the propensity to emigrate in all clusters of migratory patterns, with the effect being stronger in the ‘early return’ and ‘late return’ clusters compared to the ‘return and onward cluster’.

**Fig. 3:**
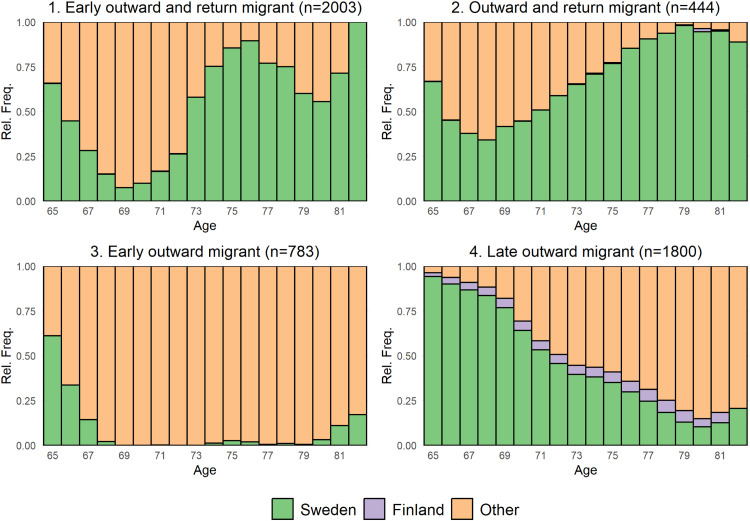
Density plots of the four migration trajectory clusters of country of residence for the Finnish born migrants that emigrated post-retirement. Note: Swedes that remained in Sweden until they died or the end of follow-up (*n* = 1708,803) are not represented.

The analyses of post-retirement migration patterns using sequence analyses among the native-born that emigrated revealed four clusters (Fig. 3). The 1708,803 individuals (99.7 %) that remained in Sweden throughout the observation period are not represented in the graph.

Clusters 1: early outward and return migrant (*N* = 2003, 39.8 %) and 2: outward and return migrant (*N* = 444, 8.8 %) represent post-retirement migration pattern with return migration to Sweden, while clusters 3: early outward migrant (*N* = 783, 15.6 %) and 4: late outward migrant (*N* = 1800, 35.8 %) represent emigration where individuals tend to stay in the new country until advanced ages. Among the native-born, cluster 4 seems to represent those that migrate to Finland. In Table S3 we present the corresponding results from a multinomial regression model for the native-born. Similar to the Finnish migrants, ill-health decreased the propensity to emigrate in all four clusters, with estimates even lower than for the Finnish migrants, suggesting a stronger effect of positive selection for the native born.

Both natives and Finnish migrants that emigrate post retirement show diverse and dynamic migration patterns. Although most tend to emigrate once and remain (either in Finland or in another country), it is also common to have several migration events, both returning to Sweden and migrating to other countries. The different migration patterns differ in their sociodemographic composition. Finnish migrants in the ‘return and onward’ cluster tend to be more similar to the native Swedes that migrate post retirement and are more likely to be men, unmarried and have a slightly higher SES than the other groups. The early and late return clusters are also more likely to be men, but no discernible effect of marital status, and with a strong reverse social gradient where the lower educated and lower income migrants are more likely to return to Finland. This reverse gradient stands in strong contrast to the native born where higher education predicts emigration in all four clusters. The income patterns are slightly different for the native born where the lowest and highest quintile are the most likely to emigrate post-retirement.

## 4 Discussion

This study used data on inpatient care in Sweden and pension payments paid abroad to (1) investigate health selection in post-retirement emigration, (2) compare mortality differentials between Finnish migrants that stay in Sweden compared to those that return migrate, and (3) evaluate sequence patterns of post-retirement migratory flows. We show that Finnish migrants have modestly higher post-retirement emigration rates than the native born, driven mostly by return migration to Finland, with three identifiable clusters: the early return migrant, the late return migrant, and the return and onward migrant. Contrary to the salmon bias hypothesis, we found ill health to decrease the likelihood of return migration, and no discernible mortality differentials between Finnish migrants that returned to Finland and those who remained in Sweden. However, after adjustment for sociodemographic factors the Finnish migrants that returned to Finland had lower mortality risk ratios than those that remained in Sweden. These finding indicate a positive health selection for return migration. Although the finding that the effect on mortality differentials is negligible is consistent with earlier studies from the US, France, Germany, and Denmark, the positive health selection found in return migration stand in contrast to studies in the US and France (Guillot et al., 2023; Turra and Elo, 2008) which find higher mortality in return migrants, while being consistent with previous studies from some Scandinavian countries and Germany (Dunlavy et al., 2022; Norredam et al., 2014). These seemingly conflicting results are likely to follow from the different contexts studied. For example, while the US provides little or no social safety nets, Finland and Sweden both have generous welfare systems that ensures universal health coverage for all residents.

The post-retirement migration patterns that emerged from sequence analysis revealed that there is some return migration to Finland after retirement, a pattern that is also seen in the native born that emigrate but then return to Sweden. The propensity to return migrate among Finnish migrants was higher among men, the childless, and those with lowest income and compulsory education. However, the rates of return migration are very small in comparison to the majority (97 %) that stay in Sweden throughout retirement. Furthermore, mortality differentials between Finnish migrants and natives did not change when extending the mortality follow-up to include deaths abroad, as opposed to censoring at emigration. One possible explanation for the low post-retirement migration rates is that migration is most common in early adulthood, so by the time a migrant reaches retirement age they have stayed for extended periods and are most likely quite settled in the host country of Sweden.

It has also been observed that the healthy migrant effect varies by age and is most pronounced in early adulthood when most migration occurs and is not as pronounced at older ages when ill-health and mortality risks increase (Boulogne et al., 2012; Handlos et al., 2018). The comparably long duration of residence for the Finnish migrants also contribute to the low rates of return migration. Slight differences in the socioeconomic predictors for return and onward migrant trajectories are found. Return migration is generally associated with lower socioeconomic status, perhaps to seek social support from family back home, while onward migration is more prevalent among pensioners with higher socioeconomic status, possibly to enjoy retirement in warmer climates (Öberg et al., 1993).

### 4.1 Methodological considerations

Receiving pension abroad is contingent on registering the move abroad. We are therefore unable to account for unregistered emigration. This is likely not an issue for Finnish return migrants since the incentives to register as resident in Finland is high since it is a prerequisite for health and social services access (Weber and Saarela, 2019), but could present an issue for using pension data to follow mortality rates for other migrant groups.

We focused our analyses on post-retirement selective migration, and our findings thus do not generalize to younger ages. A Swedish study which examined patterns of return and onwards migration for different migrant groups in Sweden at all ages find much higher emigration rates than what we observe. within particular, Nordic-born migrants - out of which Finns are the largest group - have the highest rates of return migration (Monti, 2020). Although return migration at advanced ages is less frequent, severe health complications are more common than at younger ages, thus justifying our focus and the significance of analyzing health selective migration at post-retirement ages.

A further limitation is that the register data do not contain information on several factors that may influence return migration decisions after retirement—such as exchange rates, social ties, or preferences for climate and geography—and therefore these potentially important motivations cannot be directly accounted for in the analyses.

## 5 Conclusion

Return and onward migration flows were relatively modest among Finnish migrants in Sweden, and we found no evidence of a salmon bias effect. On the contrary, poorer health reduced the likelihood of emigration in retirement, a pattern also observed among the native-born, reinforcing the notion of positive health selection in migration. Negative health selection in return migration could therefore be expected to widen, rather than narrow, mortality disparities; however, the magnitude of such flows is too limited to have a meaningful impact. The combination of administrative registers and pension payment data provide a powerful tool for studying old-age mortality and migration across national contexts as they enable a follow-up of migrants before and after emigration.

## Ethical approval

The study was approved by the Central Ethics Review Board of Sweden (Dnr Ö 25–2017).

## Study funding

This work was supported by the Swedish Research Council (grant 2021-01728). P.M. was additionally supported by the 10.13039/501100000781European Research Council under the European Union’s Horizon 2020 research and innovation programme (grant agreement No 101019329), the 10.13039/501100009047Strategic Research Council (SRC) within the Research Council of Finland grants for ACElife (#352543-352572) and LIFECON (#345219), and grants to the Max Planck—University of Helsinki Center from the Jane and Aatos Erkko Foundation (#210046), the Max Planck Society (# 5714240218).

## CRediT authorship contribution statement

**Agneta Cederström:** Writing – review & editing, Writing – original draft, Methodology, Investigation, Formal analysis, Data curation, Conceptualization. **Kaarina Korhonen:** Writing – review & editing, Conceptualization. **Pekka Martikainen:** Writing – review & editing, Methodology, Conceptualization. **Olof M Östergren:** Writing – review & editing, Writing – original draft, Project administration, Methodology, Investigation, Funding acquisition, Conceptualization.

## Declaration of competing interest

No conflict of interest declared.

## Appendix Supplementary materials

Image, application 1
