# Supplementary material for: Using pension payments abroad to examine post-retirement migration and health among Finnish migrants in Sweden
Source: J Migr Health. 2026 Mar 31;13:100411. doi: 10.1016/j.jmh.2026.100411 (PMC13089063; doi:10.1016/j.jmh.2026.100411)
Supplement: Supplementary file 1 [file mmc1.docx]

**Supplementary materials**

**Table S1.** Comparing mortality IRR between 1) study population residing in Sweden and censoring those that emigrated and 2) including those that have emigrated but death date known from pension records. Model 1 adjusts for age and sex, while Model 2 additionally adjusts for other sociodemographic factors.

Model 1

Model 2

Men

Women

Men

Women

1)

Sweden

1(ref)

1(ref)

1(ref)

Finland

1.450 (1.411-1.490)

1.209 (1.176-1.243)

1.257 (1.229-1.285)

1.111 (1.085-1.139)

2)

Sweden

1(ref)

1(ref)

1(ref)

1(ref)

Finland

1.451 (1.412-1.490)

1.206 (1.173-1.239)

1.250 (1.223-1.278)

1.106 (1.080-1.133)

**Table S2.** Demographic, socioeconomic and health-related predictors of post retirement migration patterns among Finnish migrants in Sweden. Multinomial regression. CCI and duration of residence measured at 65 years of age.

|  | Cluster 1 ref(0): Return and onward migrant | Cluster 2 ref(0): Early return migrant | Cluster 3 ref(0): Late return migrant |
| --- | --- | --- | --- |
| N | 1521 | 560 | 307 |
|  | RR(95%) | RR(95%) | RR(95%) |
| CCI |  |  |  |
| None | 1(ref) | 1(ref) | 1(ref) |
| Low | 0.78 (0.60-1.01) | 0.39 (0.23-0.68) | 0.31 (0.14-0.70) |
| High | 0.14 (0.05-0.45) | 0.13 (0.02-0.80) | - |
| Sex |  |  |  |
| Woman | 1(ref) | 1(ref) | 1(ref) |
| Man | 1.52 (1.36-1.70) | 1.40 (1.17-1.67) | 1.40 (1.10-1.77) |
| Marital status |  |  |  |
| Single | 1(ref) | 1(ref) | 1(ref) |
| Married | 0.81 (0.72-0.90) | 1.07 (0.90-1.28) | 1.00 (0.79-1.26) |
| Number of children |  |  |  |
| 0 | 1(ref) | 1(ref) | 1(ref) |
| 1 | 0.60 (0.51-0.70) | 0.44 (0.33-0.57) | 0.57 (0.40-0.80) |
| 2+ | 0.43 (0.37-0.49) | 0.36 (0.29-0.45) | 0.47 (0.35-0.62) |
| Education |  |  |  |
| Compulsory | 1(ref) | 1(ref) | 1(ref) |
| Intermediate | 0.88 (0.78-0.99) | 0.62 (0.51-0.75) | 0.65 (0.51-0.83) |
| Tertiary | 1.10 (0.94-1.30) | 0.68 (0.51-0.89) | 0.59 (0.40-0.87) |
| Disposable income |  |  |  |
| Q1(lowest) | 1(ref) | 1(ref) | 1(ref) |
| Q2 | 0.78 (0.67-0.91) | 1.14 (0.91-1.43) | 1.07 (0.80-1.42) |
| Q3 | 0.78 (0.67-0.92) | 1.04 (0.81-1.32) | 0.85 (0.62-1.17) |
| Q4 | 0.82 (0.70-0.97) | 0.65 (0.48-0.88) | 0.46 (0.30-0.71) |
| Q5(highest) | 0.86 (0.71-1.05) | 0.55 (0.37-0.83) | 0.40 (0.23-0.72) |
| Duration of residence |  |  |  |
| 0-15 | 1(ref) | 1(ref) | 1(ref) |
| 16-30 | 0.32 (0.23-0.45) | 0.38 (0.25-0.58) | 0.43 (0.23-0.82) |
| 30+ | 0.09 (0.06-0.12) | 0.05 (0.03-0.07) | 0.07 (0.04-0.14) |

**Table S3.** Demographic, socioeconomic and health-related predictors of post retirement migration patterns among the native born. Multinomial regression. CCI measured at 65 years of age.

|  | Cluster 1 ref(0): Early onward and return migrant | Cluster 2 ref(0): Onward and return migrant | Cluster 3 ref(0): Early onward migrant | Cluster 4 ref(0): Late onward migrant |
| --- | --- | --- | --- | --- |
| N | 2003 | 444 | 783 | 1800 |
|  | RR(95%) | RR(95%) | RR(95%) | RR(95%) |
| CCI |  |  |  |  |
| None | 1(ref) | 1(ref) | 1(ref) | 1(ref) |
| Low | 0.60 (0.45-0.80) | 0.19 (0.07-0.53) | 0.22 (0.11-0.46) | 0.54 (0.40-0.73) |
| High | 0.36 (0.17-0.77) | - | 0.01 (0.0-51.0) | 0.07 (0.01-0.39) |
| Sex |  |  |  |  |
| Woman | 1(ref) | 1(ref) | 1(ref) | 1(ref) |
| Man | 1.95 (1.76-2.16) | 2.52 (2.02-3.13) | 2.31 (1.96-2.72) | 2.49 (2.24-2.78) |
| Marital status |  |  |  |  |
| Single | 1(ref) | 1(ref) | 1(ref) | 1(ref) |
| Married | 0.79 (0.72-0.88) | 1.04 (0.84-1.28) | 0.91 (0.78-1.06) | 0.76 (0.69-0.84) |
| Number of children |  |  |  |  |
| 0 | 1(ref) | 1(ref) | 1(ref) | 1(ref) |
| 1 | 1.12 (0.95-1.32) | 1.59 (1.04-2.44) | 1.04 (0.81-1.33) | 1.33 (1.13-1.58) |
| 2+ | 0.92 (0.80-1.05) | 1.92 (1.34-2.77) | 0.79 (0.65-0.98) | 1.03 (0.89-1.19) |
| Education |  |  |  |  |
| Compulsory | 1(ref) | 1(ref) | 1(ref) | 1(ref) |
| Intermediate | 1.70 (1.48-1.94) | 1.12 (0.87-1.45) | 1.26 (1.03-1.53) | 1.47 (1.29-1.67) |
| Tertiary | 2.38 (2.05-2.76) | 2.37 (1.79-3.13) | 2.53 (2.04-3.13) | 2.47 (2.14-2.85) |
| Disposable income |  |  |  |  |
| Q1(lowest) | 1(ref) | 1(ref) | 1(ref) | 1(ref) |
| Q2 | 0.55 (0.46-0.65) | 0.64 (0.47-0.88) | 0.41 (0.32-0.53) | 0.59 (0.50-0.68) |
| Q3 | 0.43 (0.36-0.52) | 0.43 (0.30-0.59) | 0.31 (0.24-0.41) | 0.50 (0.43-0.59) |
| Q4 | 0.60 (0.51-0.71) | 0.37 (0.26-0.51) | 0.34 (0.27-0.43) | 0.48 (0.41-0.56) |
| Q5(highest) | 1.36 (1.18-1.57) | 0.72 (0.54-0.96) | 0.61 (0.50-0.76) | 0.67 (0.58-0.78) |
